# Supplementary material for: Revealing Impacts of Human Activities and Natural Factors on Dynamic Changes of Relationships among Ecosystem Services: A Case Study in the Huang-Huai-Hai Plain, China
Source: Int J Environ Res Public Health. 2022 Aug 17;19(16):10230. doi: 10.3390/ijerph191610230 (PMC9407877; doi:10.3390/ijerph191610230)
Supplement: Supplementary file 1 [file ijerph-19-10230-s001.zip › ijerph-1832032-supplementary.pdf]

Table S1. Description of selected landscape metrics at landscape level.

Abbreviation	Metric	Equation	Meaning of the landscape metric
SHDI	Shannon' Diversity index	$SHDI = -\sum_{i=1}^{m} p_i lnp_{i,}$ where m is the number of landscape patch types, and P_i represents the perimeter of a patch.	Reflects the richness and complexity of landscape
CONTAG	Contagion	$CONTAG = 1 + \sum_{i=1}^{m}\sum_{j=1}^{n}\frac{p_{ij}\ln(p_{ij})}{2\ln(m)},$ where P_{ij} represents the perimeter of a patch.	Reflects the spread degree of patches
PD	Patch density	$PD = \frac{NP}{A},$ where NP is the number of patches, A is the total area.	Reflects the fragmentation of landscape
LSI	Landscape shape index	$LSI = \frac{0.25L}{\sqrt{A}},$ where L is the circumference of plaque.	Reflects the complexity of landscape shape
PAFRAC	Perimeter-Area Fractal Dimension	$PAFRAC = \frac{\frac{2}{N\sum_{i=1}^{m}\sum_{j=1}^{n} lnp_{ij}lna_{ij} - \sum_{i=1}^{m}\sum_{j=1}^{n} lnp_{ij}\sum_{i=1}^{m}\sum_{j=1}^{n} lna_{ij}}}{N\sum_{i=1}^{m}\sum_{j=1}^{n} lnp^2{}_{ij} - \sum_{i=1}^{m}\sum_{j=1}^{n} lnp_{ij}},$ where a_{ij} represents the area of a patch.	Reflects the complexity of landscape shape

2.3.3. Soil conservation

R is rainfall erosion force factor (MJ mm hm^{-2} h^{-1}), the equation is as follows [1]:

$$R = \sum_{i=1}^{12} 1.735 \times 10^{\left(1.5 \times lg\frac{P_i^2}{Pa} - 0.8188\right)}, \quad (S1)$$

Where P_i is the total precipitation in month i (mm); P_a is the annual rainfall (mm).

K is soil erodibility factor (t h MJ^{-1} mm^{-1}), the equations are as follows [2]:

$$K = \left\{0.2 + 0.3e^{\left[-0.0256Sa\left(1-\frac{S_i}{100}\right)\right]}\right\} \times \left(\frac{S_i}{C_i+S_i}\right)^{0.3} \times \left\{1.0 - \frac{0.25C}{\left[C+e^{(3.72-0.95C)}\right]}\right\} \times \left\{1.0 - \frac{0.7\times\left(1-\frac{Sa}{100}\right)}{\left(1-\frac{Sa}{100}\right)+e^{\left(-5.51+22.9\times\left(1-\frac{Sa}{100}\right)\right)}}\right\}, \quad (S2)$$

Where S_a, S_i, C_i and C denote the content of sand, silt, clay, and organic matter in the soil, respectively.

L is slope length factor, the equations are as follows [3]:

$$L = \left(\frac{\gamma}{22.13}\right)^m, \quad (S3)$$

$$m = \frac{n}{n+1}, \quad (S4)$$

$$n = \frac{\sin(\theta)}{0.0896 \times (3 \times (sin\theta)^{0.8} + 0.56)}, \quad (S5)$$

Where θ is slope angle; γ is regional cumulative sink flow.

S is slope factor, the equations are as follows [3]:

$$S = 10.8 \times \sin\theta + 0.03, \theta < 5°, \quad (S6)$$

$$S = 16.8 \times \sin\theta - 0.5, 5° \le \theta < 10°, \quad (S7)$$

$$S = 21.9 \times \sin\theta - 0.96, \theta \ge 10°, \quad (S8)$$

C is vegetation cover factor, the calculation methods are as follows [4]:

$$C = \begin{cases} 1, & f = 0 \\ 0.6508 - 0.3436 \times \lg(f), & 0 < f < 78.3\%, \\ 0, & f \ge 78.3\% \end{cases} \quad (S9)$$

$$f = \frac{(NDVI - NDVI_{soil})}{(NDVI_{max} - NDVI_{soil})}, \quad (S10)$$

Where f represents the vegetation cover.

P is soil and water conservation factor and its value referred to [5].

Table S2. *p* values of different land use types in the HHHP.

LULC	P
Forest land	1
Grassland	1
Water area	0
Urban land	0
Paddy field	0.35
Dry land	0.6
Unused land	1

2.3.4. Water yield

AWC_X indicates the available water of vegetation (mm), the equation are as follows [6, 7]:

$$AWC_X = \min(Soildepth_x, Rootdepth_x) \times PAWC_x, \quad (S11)$$

$$Soildepth_x = \text{REF\_DEPTH} \times 10, \quad (S12)$$

$$PAWC = 0.301 \times \text{clay\%} + 0.369 \times \text{silt\%} + 0.045 \times \text{om\%}, \quad (S13)$$

Where $PAWC_x$, $Soildepth_x$, $Rootdepth_x$ is the effective soil water content, soil depth and plant root depth of image x, respectively; REF_DEPTHD is a field in the soil database; Clay (%) is the percentage content of clay, silt (%) is the percentage content of chalk, om (%) is the percentage content of organic matter.

Z is a seasonal constant; Formulas are as follows:

$$Z = \frac{(w - 1.25)^P}{AWC}, \quad (S14)$$

$$AWC = \text{PAWC} \times \text{REF\_DEPTH} \times 10, \quad (S15)$$

$$w = 0.69387 - 0.01042lat + 2.81063NDVI + 0.146186CTI, \quad (S16)$$

Where P is the average annual precipitation, AWC is the average annual plant available water; *Lat* is the absolute dimension of the watershed, *NDVI* is the multi-year average normalized vegetation index of the watershed, and *CTI* is the composite topographic index.

ETo_x is the reference vegetation evapotranspiration of grid x (mm), the equation is as follows [8]:

$$ETo_x = 0.0013 \times 0.408 \times \mathrm{RA} \times (\mathrm{Tav} + 17) \times (TD - 0.0123P)^{0.76}, \quad (S17)$$

Where *TD* is the average value of the difference between the daily maximum and minimum temperature in a year; *RA* is the total monthly radiation; *Tav* is the sum of the maximum and maximum temperature on a monthly scale divided by 2; and P is the average monthly precipitation in a year.

Biophysical coefficients required by the WY model can be found in Table S3.

Table S3. Parameter values of biophysical coefficient for WY module

LULC	Lucode	LULC_Veg	Root_depth	Kc_x
Forest land	1	1	3500	1
Grassland	2	1	2400	0.65
Water area	3	0	1000	1.1
Urban land	4	0	10	0.5
Cropland	5	1	2000	0.65
Unused land	6	0	500	0.5

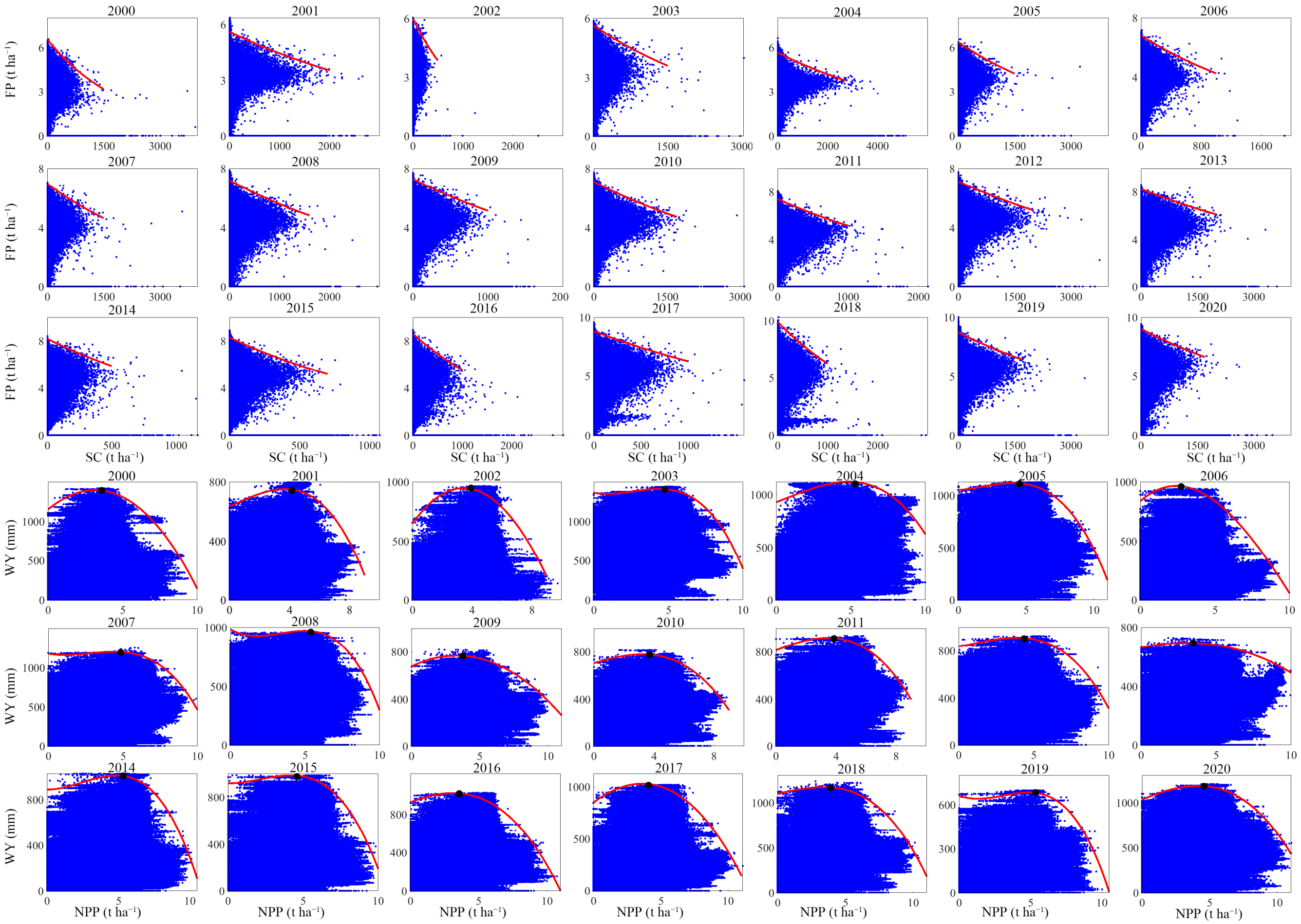

Figure S1. The scatter clouds (blue points), thresholds (black points), and constraint lines (red lines) between paired ESs (A_B) from 2000 to 2020. A indicates the constraint ES on the x-axis and B indicates the corresponding ES on the y-axis. NPP: net primary productivity; FP: food production; SC: soil conservation; WY: water yield. The units of NPP, SC, FP and WY are t ha^{-1} year^{-1}, t ha^{-1} year^{-1}, t ha^{-1} year^{-1} and mm, respectively.

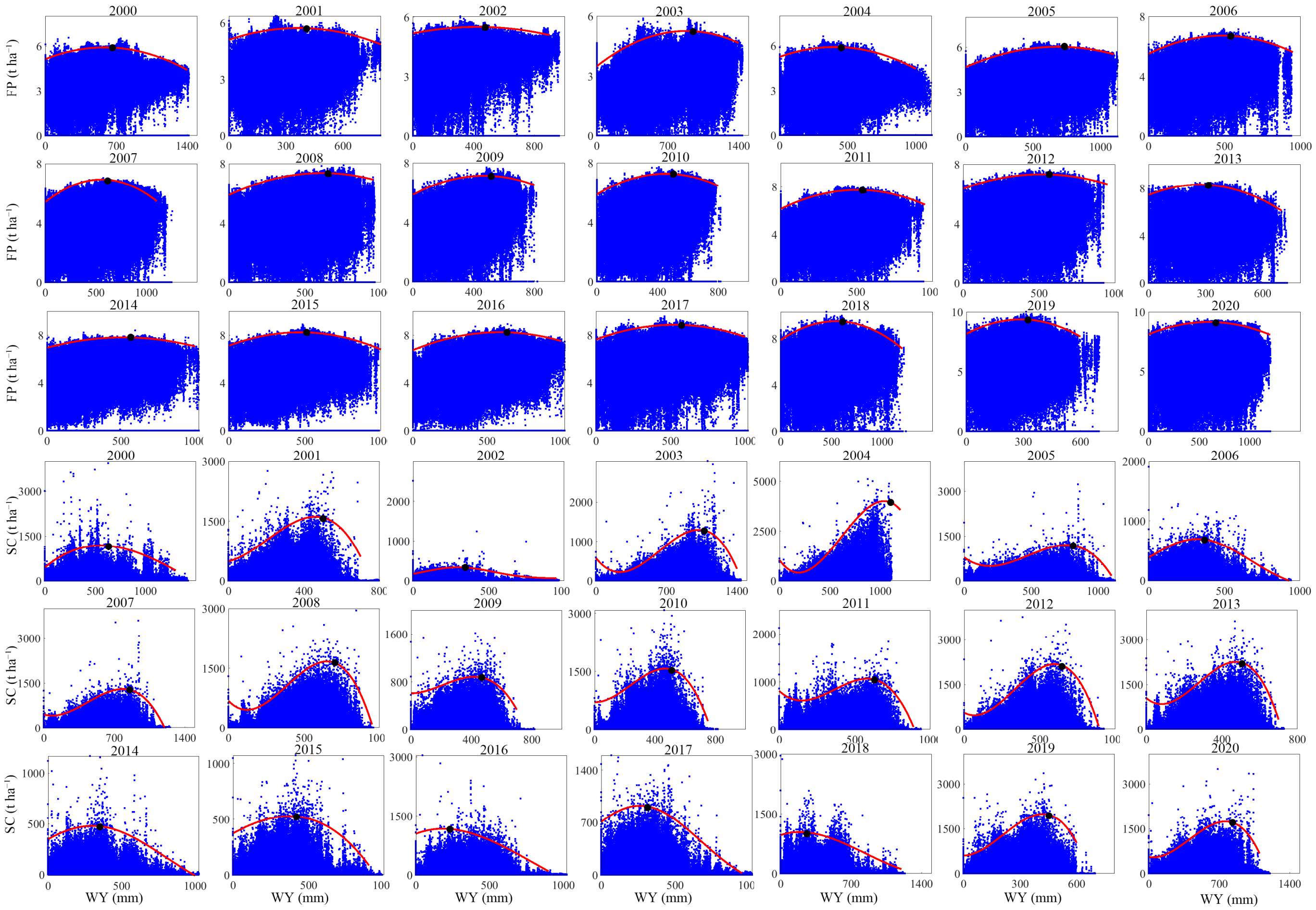

Figure S2. The scatter clouds (blue points), thresholds (black points), and constraint lines (red lines) between paired ESs (A_B) from 2000 to 2020. A indicates the constraint ES on the x-axis and B indicates the corresponding ES on the y-axis. FP: food production; SC: soil conservation; WY: water yield. The units of FP, SC and WY are t ha^{-1} year^{-1}, t ha^{-1} year^{-1} and mm, respectively.

Table S4. The functions of the constraint lines between paired ESs from 2000 to 2020 in the HHHP.

	SC_FP	FP_WY
2000	y=6.5378*0.99952^x	y=-0.000002x^2+0.002x+5.12
2001	y=5.60395*0.99977^x	y=-0.000004x^2+0.003x+5.09
2002	y=6.02053*0.99911^x	y=-0.000002x^2+0.001x+5.17
2003	y=5.62525*0.9997^x	y=-0.000002x^2+0.004x+3.48
2004	y=5.64877*0.99985^x	y=-0.000004x^2+0.004x+5.25
2005	y=6.34761*0.99973^x	y=-0.000003x^2+0.003x+4.65
2006	y=6.80031*0.99953^x	y=-0.000005x^2+0.005x+5.50
2007	y=6.95183*0.99973^x	y=-0.000005x^2+0.005x+5.41
2008	y=7.18541*0.99975^x	y=-0.000004x^2+0.004x+5.88
2009	y=7.28664*0.99965^x	y=-0.000006x^2+0.005x+5.86
2010	y=7.09159*0.99976^x	y=-0.000007x^2+0.006x+5.88
2011	y=7.39706*0.99964^x	y=-0.000006x^2+0.006x+6.12
2012	y=7.11188*0.99984^x	y=-0.000004x^2+0.003x+6.36
2013	y=8.24467*0.99985^x	y=-0.000012x^2+0.006x+7.42
2014	y=8.17883*0.99934^x	y=-0.000003x^2+0.003x+6.95
2015	y=8.28793*0.99933^x	y=-0.000005x^2+0.004x+7.12
2016	y=8.61142*0.99956^x	y=-0.000004x^2+0.005x+6.74
2017	y=8.82461*0.99966^x	y=-0.0000004x^2+0.004x+7.63
2018	y=9.91623*0.99954^x	y=-0.000005x^2+0.005x+7.87
2019	y=8.72783*0.99982^x	y=-0.000015x^2+0.008x+8.18
2020	y=9.01285*0.99982^x	y=-0.000003x^2+0.003x+8.12
	NPP_SC	NPP_WY
2000	y=-30.7x^2+230.9x+953.2	y=152.3x-23.1x^2-0.2x^3+1151.4
2001	y=-51.1x^2+478.9x+396.2	y=45.5x+1.2x^2-1.3x^3+633.2
2002	y=-5.2x^2+44.4x+237.2	y=176.2x-26.4x^2+0.1x^3+648.1
2003	y=-28.4x^2+295.3x+539.7	y=-33.3x+23x^2-2.9x^3+1360.8
2004	y=-48.1x^2+716.8x+585.4	y=54.1x+2.2x^2-1.1x^3+927.9
2005	y=-25.9x^2+252.4x+524.6	y=10.6x+6.9x^2-1.3x^3+1049.1
2006	y=-15.5x^2+145.2x+385.3	y=111.1x-25.5x^2+0.6x^3+836.5
2007	y=-28.7x^2+260.1x+623.7	y=-32.8x+18.5x^2-2.2x^3+1175.3
2008	y=-37.2x^2+321.1x+817.8	y=-72.2x+27.5x^2-2.7x^3+983.3
2009	y=-19.1x^2+201x+417.4	y=55x-8.2x^2-0.015x^3+675.8
2010	y=-68.5x^2+546.7x+535.8	y=38.4x-2.1x^2-0.7x^3+702.4
2011	y=-21.6x^2+155.9x+752.5	y=51.1x-2.8x^2-0.8x^3+812.6
2012	y=-51.7x^2+532.4x+604.3	y=22x+3.6x^2-1.1x^3+836.8
2013	y=-29.7x^2+333.1x+1061.4	y=19.7x-2.9x^2-0.07x^3+662.9
2014	y=-5.7x^2+45.3x+414.8	y=-0.8x+15.9x^2-2.1x^3+887.8
2015	y=-13.4x^2+133.6x+227	y=-1.9x+12.5x^2-1.9x^3+917.4
2016	y=-21.2x^2+150x+937.2	y=56.7x-6.5x^2-0.5x^3+925.3
2017	y=-14.8x^2+143.4x+546.5	y=95.7x-11.8x^2-0.2x^3+850.2
2018	y=-23.6x^2+220.4x+651.3	y=30.5x+0.03x^2-0.9x^3+1098.7

2019	y=-58.8x^2+584.9x+341.1	y=-36.9x+17.8x^2-1.9x^3+663.3
2020	y=-26.5x^2+267.9x+867.6	y=62.4x-3.6x^2-0.6x^3+1032.3
	NPP_FP	WY_SC
2000	y=-0.05x^2+0.59x+4.35	y=2.8x-0.003x^2+0.0000006x^3+446.6
2001	y=-0.07x^2+0.81x+3.42	y=1.1x+0.01x^2-0. 000017x^3+490.1
2002	y=-0.03x^2+0.81x+3.98	y=57.6+281.3e^ (-0.5((x-300)/(-226))^2)
2003	y=-0.02x^2+0.43x+3.57	y=-3.2x+0.008x^2-0.0000044x^3+575.7
2004	y=-0.03x^2+0.42x+4.82	y=-6.9x+0.02x^2-0.000011x^3+1034.6
2005	y=-0.02x^2+0.35x+4.53	y=-3.2x+0.01x^2-0.0000075x^3+783.2
2006	y=-0.06x^2+0.64x+5.02	y=-205.2+901.2e^(-0.5((x-321)/(-350.2))^2)
2007	y=-0.04x^2+0.56x+4.79	y=-0.9x+0.006x^2-0.0000051x^3+453.3
2008	y=-0.06x^2+0.75x+5.23	y=-3.9x+0.018x^2-0.000016x^3+681.2
2009	y=-0.04x^2+0.50x+5.51	y=-0.01x+0.004x^2-0.0000076x^3+609.4
2010	y=-0.05x^2+0.58x+5.52	y=-0.1x+0.011x^2-0.000017x^3+711
2011	y=-0.08x^2+0.90x+5.21	y=-2.9x+0.012x^2-0.000011x^3+810.6
2012	y=-0.04x^2+0.51x+5.76	y=-2.6x+0.022x^2-0.000022x^3+560.3
2013	y=-0.06x^2+0.72x+6.04	y=-5.1x+0.038x^2-0.000046x^3+1043.02
2014	y=-0.04x^2+0.59x+5.80	y=-309+788.7e^(-0.5((x-301.9)/(-500.6))^2)
2015	y=-0.03x^2+0.51x+6.28	y=0.8x-0.001x^2-0.00000053x^3+372.3
2016	y=-0.05x^2+0.62x+6.28	y=1.4x-0.004x^2+0.0000019x^3+1044.8
2017	y=-0.05x^2+0.62x+7.01	y=-267+1191e^(-0.5((x-259.7)/(-411.1))^2)
2018	y=-0.07x^2+0.83x+7.01	y=0.8x-0.0024x^2+0.00000097x^3+963.5
2019	y=-0.1x^2+0.99x+6.83	y=0.09x+0.023x^2-0.000036x^3+600.5
2020	y=-0.06x^2+0.62x+7.50	y=-0.67x+0.0079x^2-0.0000065x^3+556.9

Notes: For the paired ESs of A_B, A indicates the constraint ES on the x-axis and B indicates the corresponding ES on the y-axis. NPP: net primary productivity; FP: food production; SC: soil conservation; WY: water yield.

1. Wang, B.; Tang, H.; Xu, Y. Integrating ecosystem services and human well-being into management practices: Insights from a mountain-basin area, China. *Ecosystem Services* **2017**, *27*, 58-69.
2. Gou M , L.L., Ouyang S. Identifying and analying ecosystem service bundles and their socioecological drivers in the Three Gorges Reservoir Area. *Journal of Cleaner Production* **2021**, *307*, 127208.
3. Liu, B.Y.; Nearing, M.A.; Risse, L.M. Slope Length Effects on Soil Loss for Steep Slopes. *Soil Science Society of America Journal* **2000**, *64*, 1759-1763.
4. Li, S.K.; Li, X.B.; Dou, H.S.; Dang, D.L.; Gong, J.R. Integrating constraint effects among ecosystem services and drivers on seasonal scales into management practices. *Ecological Indicators* **2021**, *125*, 107425.
5. Xu, Y.J.; Yao, Z.H.; Zhao, D.B. Estimating Soil Erosion in North China Plain Based on

RS/GIS and RUSLE. *Bulletin of Soil and Water Conservation* **2012**, *32*, 217-221.

6. Peng, J.; Hu, X.; Wang, X.; Meersmans, J.; Liu, Y.; Qiu, S. Simulating the impact of Grain-for-Green Programme on ecosystem services trade-offs in Northwestern Yunnan, China. *Ecosystem Services* **2019**, *39*, 100998.
7. Renard, K.G.; Foster, G.R.; Weesies, G.A.; Porter, J.P. RUSLE: Revised universal soil loss equation. *J Soil & Water Conservation* **1991**, *46*, 1-9.
8. Hao, R.F.; Yu, D.Y.; Wu, J.G. Relationship between paired ecosystem services in the grassland and agro-pastoral transitional zone of China using the constraint line method. *Agriculture, Ecosystems & Environment* **2017**, *240*, 171-181.
